# Activity is a slave to many masters

**DOI:** 10.7554/eLife.06351

**Published:** 2015-02-12

**Authors:** Andrew D Steele, Ralph E Mistlberger

**Affiliations:** Department of Biological Sciences, California State Polytechnic University Pomona, Pomona, United States, adsteele@cpp.edu; Department of Psychology, Simon Fraser University, Burnaby, Canada, mistlber@sfu.ca

**Keywords:** chronobiology, dopamine, circadian rhythms, ultradian, oscillator, dopamine transporter, Mouse

## Abstract

Dopamine neurons in the midbrain have a central role in generating cycles of biological activity with periods as short as 4 hours and as long as 100 hours.

**Related research article** Blum ID, Zhu L, Moquin L, Kokoeva MV, Gratton A, Giros B, Storch KF. 2014. A highly-tunable dopaminergic oscillator generates ultradian rhythms of behavioral arousal. *eLife* **3**:e05105. doi: 10.7554/eLife.05105

**Image** Methamphetamine increases the period of ultradian activity rhythms in Bmal1 null mice by increasing dopamine levels

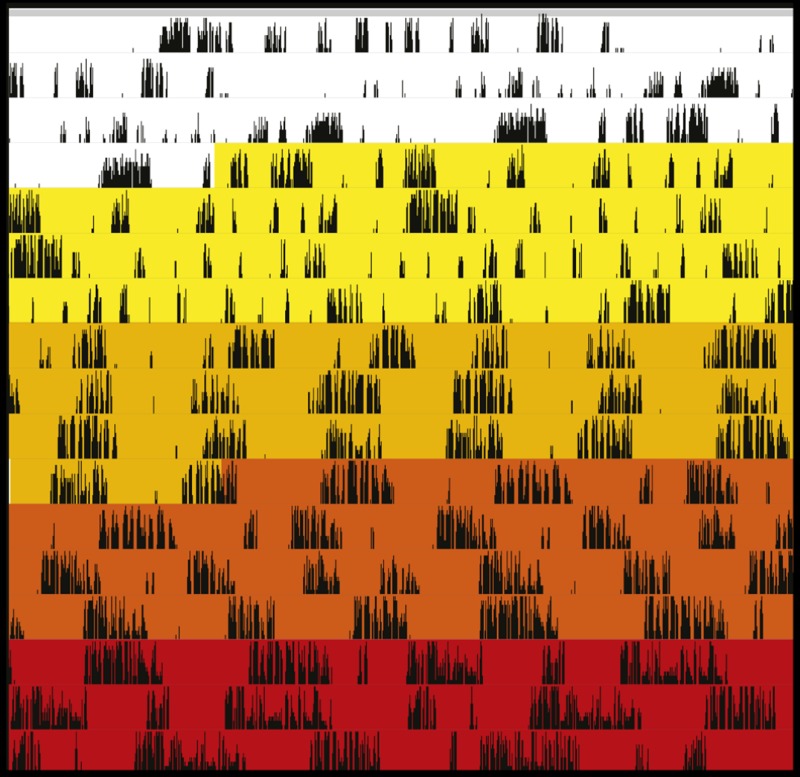

The rotation of the earth as it orbits around the sun creates daily cycles of light, temperature and other factors that are vitally important for myriad biological processes. Indeed, almost all organisms exhibit circadian gene expression—that is, certain genes are expressed as proteins at a rate that oscillates with a period of about 24 hr—and associated biochemical and behavioral rhythms. Circadian rhythms are the organization of biological activities into cycles that oscillate with a period of around 24 hours, and we now know a great deal about the molecular and neural bases of these rhythms (Bell-Pedersen et al., 2005; Mohawk et al., 2012). Moreover, the disruption of circadian rhythms by neural, genetic or environmental perturbations is associated with a range of disease processes and increased mortality (Evans and Davidson, 2013).

Animal behavior and physiology also exhibit rhythms than that have periods that are substantially less than (i.e., ‘ultradian’) or greater than (i.e., ‘infradian’) 24 hours. The underlying mechanisms for most of these rhythms are poorly understood. Now, in *eLife*, Ian Blum and Kai-Florian Storch of McGill University and the Douglas Institute, both in Montreal, and co-workers report that dopamine—a chemical in the brain that is usually associated with pleasure and motivated reward—has a central role in setting the frequency of ultradian rhythms and may also account for rhythmic phenomena with longer periods in the circadian and infradian ranges (Blum et al., 2014).

In mammals, circadian rhythms can be eliminated by ablation of the suprachiasmatic nucleus (SCN) in the brain or by the deletion of so-called circadian clock genes. The starting point for Blum et al. was the observation that mice lacking a circadian rhythm because their SCN had been ablated, or because the clock gene *Bmal1* had been deleted, still maintained an activity rhythm with a period of approximately 4 hours. The link to dopamine was made when they tested mice lacking both the SCN and the dopamine reuptake transporter (a protein that decreases dopamine levels at neuronal synapses). In these mice, ultradian rhythms lengthened from 4 hours to 12 hours. A similar effect was obtained by administering methamphetamine, a stimulant that also increases synaptic dopamine: indeed, the period increased with the dose, reaching 100 hours at the highest doses.

To directly test the hypothesis that ultradian rhythms are driven by a ‘dopamine ultradian oscillator’, Blum et al. measured the level of dopamine in several contexts. They found that the dopamine level in a region of the brain called the dorsal striatum rose and fell in synchrony with the ultradian rhythm. Moreover, they used a chemogenetic approach to directly manipulate the dopamine neurons in the midbrain of mice in which the SCN had been ablated and the *Bmal1* gene had been deleted. They found, as expected, that increased activation of dopamine neurons increased the period of the ultradian rhythms. These data reveal for the first time a neurochemical that regulates the rate at which ultradian rhythms cycle. From these observations, Blum et al. speculate that dysregulation of dopamine signaling in the human brain could amplify and lengthen ultradian rhythms, resulting in cycles of mood and motivation with periods of 24 hours or longer, such as those characteristic of depression and bipolar disorder.

The dopamine ultradian oscillator concept may also be relevant to two other rhythms that operate independently of the circadian pacemaker in the SCN. One of these is the effect of methamphetamine: as mentioned above, high doses of this stimulant induce infradian rhythms that uncouple from the circadian rhythm in wild-type rodents. These rhythms, previously attributed to a ‘methamphetamine sensitive circadian oscillator’ (Tataroglu et al., 2006), can now be understood as the output of dopamine ultradian oscillators: these oscillators are presumably normally coupled to the circadian pacemaker in the SCN, but high levels of dopamine may increase their period and drive them out of synchrony with the SCN pacemaker (Figure 1).

**Figure 1.**
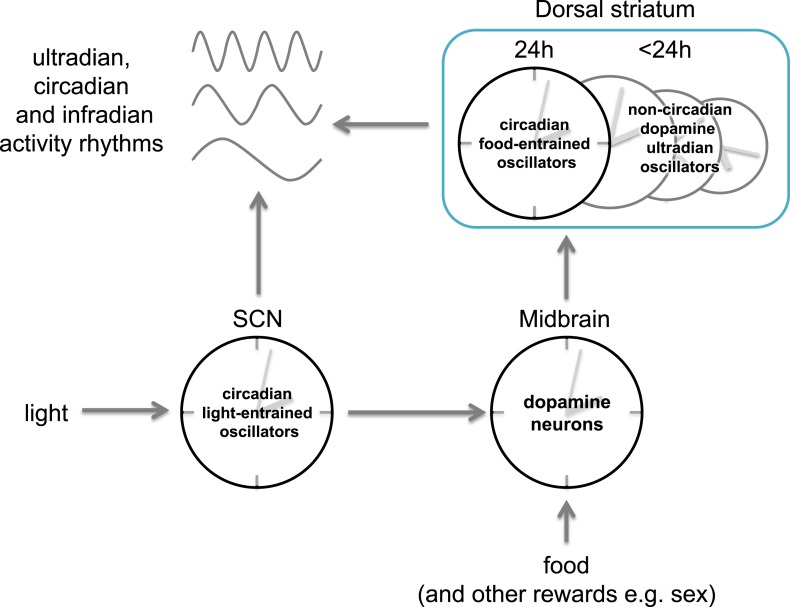
Different kinds of body clocks. Retinal input to a clock in the suprachiasmatic nucleus (SCN) entrains circadian (∼24 hr) rhythms to daily cycles of light and darkness. Dopamine neurons in the midbrain and dopamine sensitive neurons in the dorsal striatum appear to be crucial for regulating activity rhythms with periods of ~4 hours: these ultradian oscillators can operate independently of the SCN clock, and can be driven to periodicities in the 12-100 hour range under high dopaminergic tone. Some of these oscillators may be constrained to circadian periods and control activity rhythms that anticipate daily meals or other rewards that activate dopamine neurons at ∼24-hr intervals. Daily variations in activity may therefore reflect joint control by ultradian and circadian oscillators under dopamine control.

The second effect concerns the rhythms of food anticipatory activity that emerge in mice, rats and other species when food access is restricted to a fixed time of day (Mistlberger, 2011). These rhythms exhibit all the properties of circadian clock control, but they do not require a SCN or the clock gene *Bmal1*. The search for the neural substrates of these food anticipatory rhythms has focused on the hypothalamus, but there is growing evidence that dopamine neurons in the midbrain may be important. This was first suggested by observations that circadian anticipatory rhythms can be induced by restricted access to sex, water or palatable treats. The dopamine neurons in the midbrain project to the ventral and dorsal striatum, and both of these areas exhibit circadian rhythms in the expression of clock genes (Guilding and Piggins, 2007). In the dorsal striatum, the clock gene rhythm is eliminated by dopamine depletion and shifted by dopamine receptor agonists (Hood et al., 2010). Notably, rhythms in both areas are synchronized by restricted feeding schedules, and restoration of dopamine signaling in the dorsal striatum is sufficient to rescue food anticipatory rhythms in dopamine-deficient mice (Gallardo et al., 2014). These and other results demonstrate that food anticipatory rhythms, like ultradian rhythms, are dopamine sensitive and require neither the SCN nor the clock gene Bmal1.

These similarities invite speculation that the ultradian and food anticipatory rhythms share a common mechanism. One argument against this idea is that the latter exhibit mostly circadian constraints: that is, they operate best at cycles close to 24 hours). However, the circadian constraint is lost in *Bmal1* knockout mice, which can anticipate meals across a much broader range of intervals (Takasu et al., 2012). These findings can be accommodated by a model in which temporal control of rest–activity cycles is mediated by a heterogenous population of oscillators that cycle at different frequencies (Figure 1).

Some of these oscillators are not constrained by circadian clock genes, and they regulate ultradian cycles of foraging (and other activities) that can lengthen to infradian cycles under high levels of dopamine. Other oscillators are constrained by circadian clock genes and control circadian cycles of foraging activity when food availability recurs predictably at 24-hour intervals. All of these oscillators may be dopamine sensitive, and conceivably may reside in midbrain dopamine neurons, the dorsal striatum, or both.

The same population of oscillators may also be involved in other manifestations of timing behavior, such as the ability to sense the passage of time in the seconds to minutes range (so-called ‘interval timing’). Impaired time perception is another manifestation of psychiatric disorders, which leads back to the suggestion by Blum et al. that dopamine ultradian oscillators in the striatum may be critical for understanding mood cycling and thought disorders.

What remains to be established is whether the timing mechanisms responsible for ultradian rhythms are located entirely within midbrain dopamine neurons and the dorsal striatum, or are distributed more broadly, and how these mechanisms operate at the molecular level. From these answers may emerge new treatments for disorders of temporal organization that may underpin mental illness.
